# The Value of a Comprehensive Genomic Evaluation in Prenatal Diagnosis of Genetic Diseases: A Retrospective Study

**DOI:** 10.3390/genes13122365

**Published:** 2022-12-14

**Authors:** Fang Fu, Ru Li, Qiu-Xia Yu, Xiao Dang, Shu-Juan Yan, Hang Zhou, Ken Cheng, Rui-Bin Huang, You Wang, Yong-Ling Zhang, Xiang-Yi Jing, Li-Na Zhang, Dong-Zhi Li, Can Liao

**Affiliations:** Department of Prenatal Diagnostic Center, Guangzhou Women and Children’s Medical Center, Guangzhou Medical University, Guangzhou 510180, China

**Keywords:** copy number variants, CMA, exome sequencing, CNV-seq, prenatal diagnosis

## Abstract

Currently, there are still many challenges in prenatal diagnosis, such as limited or uncertain fetal phenotyping, variant interpretation, and rapid turnaround times. The aim of this study was to illustrate the value of a comprehensive genomic evaluation in prenatal diagnosis. We retrospectively reviewed 20 fetuses with clinically significant copy number variants (CNVs) detected by chromosomal microarray analysis (CMA) and no further exome sequencing testing in our tertiary center between 2019 and 2020. The residual DNA from the prenatal cases was used for the parallel implementation of CNV sequencing (CNV-seq) and trio-based clinical exome sequencing (trio-CES). CMA revealed 26 clinically significant CNVs (18 deletions and eight duplications) in 20 fetuses, in which five fetuses had two or more CNVs. There were eight fetuses with pathogenic CNVs (e.g., del 1p36), nine fetuses with likely pathogenic CNVs (e.g., dup 22q11.21), and three fetuses with variants of unknown significance (VOUS, e.g., dup 1q21.1q21.2). Trio-CES identified four fetuses with likely pathogenic mutations (SNV/InDels). Of note, a fetus was detected with a maternally inherited hemizygous variant in the *SLX4* gene due to a 16p13.3 deletion on the paternal chromosome. The sizes of CNVs detected by CNV-seq were slightly larger than that of the SNP array, and four cases with mosaic CNVs were all identified by CNV-seq. In conclusion, microdeletion/duplication syndromes and monogenic disorders may co-exist in a subject, and CNV deletion may contribute to uncovering additional recessive disease alleles. The application of a comprehensive genomic evaluation (CNVs and SNV/InDels) has great value in the prenatal diagnosis arena. CNV-seq based on NGS technology is a reliable and a cost-effective technique for identifying CNVs.

## 1. Introduction

Fetal structural anomalies are relatively frequent findings in routine prenatal ultrasonographic examinations, which might serve as clues to the underlying molecular genetic abnormalities. Different types of genomic variations are associated with genetic disorders, ranging from single nucleotide variations (SNVs) to small and large-scale chromosome structural variations. In the prenatal setting, conventional karyotyping (G-banding) is used for the analysis of chromosomal rearrangements with a resolution at 5–10 Mb. High-resolution chromosome microarray analysis (CMA) is the gold standard for detecting submicroscopic copy number variants (CNVs) in fetuses with a normal karyotype. If karyotype analysis and CMA have failed to yield a definitive diagnosis, exome sequencing (ES) is recommended for the identification of potential monogenic disorders [1,2,3].

Compared with the CMA, the advantages of exome sequencing are: deeper coverage; higher data accuracy; more simple, economical, and efficient. For some difficult and complicated diseases that have atypical characteristics and cannot be diagnosed clinically, exome sequencing detection can clarify the cause of the disease and assist the diagnosis. In prenatal diagnosis, exome sequencing measures a wide range and may detect more severe VOUS, which is not easy to interpret in clinical genetic counseling. It may be accompanied by related ethical issues about whether to report and whether to terminate the pregnancy. Since the probe sequence is designed according to the target sequence and is deterministic, the CMA can only detect known sequences.

CMA includes array comparative genomic hybridization (aCGH) and single nucleotide polymorphism (SNP) arrays, which provides an additional diagnostic yield of 3% to 6% in fetuses with abnormal ultrasound findings and a normal karyotype [4,5,6,7]. ES has been applied in prenatal diagnosis to identify monogenic diseases that cause fetal structural anomalies, with an overall diagnostic yield range from 10% to 33% in different cohorts [8,9,10,11]. It is a fact that one or more types of pathogenic or likely pathogenic genetic variations may exist in an individual, such as SNVs, small insertions and deletions (InDels), and CNVs. Double diagnoses or autosomal recessive (AR) genetic diseases caused by CNVs, and SNV/InDels have been reported in many postpartum or prenatal studies [12,13,14,15,16,17]. In the current mainstream sequential procedure in prenatal diagnosis, cases with clinically significant CNVs detected by CMA would not be referred for ES, which may omit the possibility of identifying other underlying monogenic diseases. However, there are still many challenges in prenatal diagnosis, such as limited or uncertain fetal phenotyping, variant interpretation, and rapid turnaround times. Recently, several studies have applied CMA or CNV sequencing (CNV-seq) and ES concurrently in fetuses with structural anomalies and a normal karyotype, which improved the overall diagnostic yield in prenatal diagnosis [18,19,20].

The SNP array is a powerful platform for detecting genome-wide CNVs, including chromosome aneuploidies, unbalanced chromosomal rearrangements, microdeletions and microduplications, uniparental isodisomy (UPD), and low level mosaicism [21,22]. Nowadays, CNV-seq based on next-generation sequencing (NGS) is emerging as an alternative methodology to CMA for the detection of clinically significant chromosomal abnormalities. The low-pass genome sequencing (<1× coverage) method is characterized by a higher resolution down to 100 kb and relatively low cost and has been gradually used in prenatal diagnosis [23,24,25]. Compared with CMA, CNV-seq is more efficient due to its smaller sample size, faster experimental cycle and lower cost.

In this study, we retrospectively reviewed 20 prenatal cases with clinically significant CNVs detected by CMA (SNP array) and no further exome sequencing testing. All the fetuses were characterized by ultrasonic anomalies or positive noninvasive prenatal testing (NIPT) results. The residual DNA from the prenatal cases was used for the parallel implementation of CNV-seq and trio-based clinical exome sequencing (trio-CES). In addition, the detection of CNVs by CNV-seq was compared with SNP array analysis.

## 2. Methods

### 2.1. Study Design

This study was approved by the Ethics Committee of Guangzhou Women and Children’s Medical Center (2015278B01 and 2021356B01). Written informed consent was obtained from all participating families. This retrospective case series was based on data from 20 fetuses at the Prenatal Diagnostic Center of Guangzhou Women and Children’s Medical Center from 2019 to 2020. The inclusion criteria were as follows: pregnant women with fetuses with abnormal ultrasound or NIPT findings were considered for invasive prenatal diagnosis; clinically significant CNVs were detected by SNP array and no further exome sequencing testing; the extracted DNA samples were retrieved retrospectively for CNV detection by CNV-seq and SNV/InDels detection by trio-CES; the parents of the children signed the informed consent before any test was performed.

### 2.2. Chromosome Microarray Analysis (CMA)

The genome-wide high-resolution SNP array on the CytoScan HD instrument (Afymetrix, Santa Clara, CA, USA) was performed according to the manufacturer’s protocol. The size threshold of the CNVs reporting was set to 100 kb with a marker count of ≥50. Data analyses were carried out by Chromosome Analysis Suite software (Version 3.0, Affymetrix, Santa Clara, CA, USA), and results were interpreted using public CNV databases, investigating gene content and the peer-reviewed literature. The publicly available databases included the database of genomic variants (DGV, http://dgv.tcag.ca/dgv/app/home/ (accessed on 20 June 2022)), DECIPHER (http://decipher.sanger.ac.uk/ (accessed on 20 June 2022)), the International Standards for Cytogenomic Arrays (ISCA, https://www.iscaconsortium.org/ (accessed on 20 June 2022)), the Online Mendelian Inheritance in Man (OMIM, http://www.omim.org (accessed on 20 June 2022)), and the University of California Santa Cruz (UCSC, http://genome.ucsc.edu/ (accessed on 20 June 2022)). According to the guidelines, the CNVs were classified as benign, pathogenic, or variants of unknown significance (VOUS) [26,27]. SNP array analysis was performed for all samples, and fetuses with pathogenic CNVs were tested by the CNV-seq assay. Parental blood samples were collected to exclude maternal cell contamination and to help with CNV interpretation.

### 2.3. Clinical Exome Sequencing (CES)

Custom-designed NimbleGen SeqCap probes (Roche NimbleGen, Madison, WI, USA) were used for in-solution hybridization to enrich target sequences, which included coding exons for ~5000 clinically relevant disease-causing genes. The genes were selected based on reports in OMIM, HGMD, and peer-reviewed literature. Known pathogenic variants in deep intronic and other non-coding regions in targeted genes were also included. Enriched DNA samples were indexed and sequenced on a NextSeq 500 or a NovaSeq 6000 sequencer (Illumina, San Diego, CA, USA) with 150 cycles of single end reads, as previously described [28]. The average coverage depth was 200×, with >99% of the target regions covered at >20×. Sequencing reads were mapped to the reference human genome version hg19 (2009-02 release, http://genome.ucsc.edu/ accessed on 20 June 2022). Nucleotide changes observed of aligned reads were called by the NextGENe software (Version 2.4.1.2) (SoftGenetics, State College, PA, USA). The interpretation of pathogenicity of variants was evaluated according to the American College of Medical Genetics (ACMG) guidelines [29].

### 2.4. CNV Sequencing (CNV-Seq)

Low-coverage or low-pass whole genome coverage was used to evaluate CNVs. Genomic DNA was extracted, followed by random fragmentation and short-read sequencing on the NextSeq 500. The average resolution was 100 kb. Sequencing reads were mapped to the reference human genome version hg19 (2009-02 release, http://genome.ucsc.edu/ (accessed on 23 June 2022)). CNVs were evaluated by an in-house bioinformatics pipeline according to read counts and Z-scores (AmCare Genomics Lab, Guangzhou, China). The interpretation of pathogenicity of CNVs was evaluated with in-house database and public CNV databases, including DGV (http://dgv.tcag.ca/dgv/app/home (accessed on 23 June 2022)), the DECIPHER database (http://decipher.sanger.ac.uk/ (accessed on 23 June 2022)), ClinGen (https://www.clinicalgenome.org/ (accessed on 23 June 2022)), and the Online Mendelian Inheritance in Man (OMIM, http://www.omim.org (accessed on 23 June 2022)).

## 3. Results

### 3.1. Cases Description

A total of 20 fetuses were identified as eligible for inclusion in this study, including 3 fetuses with positive NIPT results, 6 fetuses with ultrasonic soft markers, and 11 fetuses with ultrasonographic structural abnormalities. There were four chorionic villi and 16 amniotic fluid samples. All parents were asymptomatic and had an unremarkable family history.

### 3.2. Genetic Variants Detected by CMA and CES

CMA revealed 26 clinically significant CNVs (18 deletions and eight duplications) in 20 fetuses, of which 5 fetuses had two or more CNVs (Table 1). There were eight fetuses with pathogenic CNVs, nine fetuses with likely pathogenic CNVs, and three fetuses with variants of unknown significance (VOUS). Trio-CES identified four fetuses with likely pathogenic SNVs and a frameshift mutation and two fetuses with de novo or hemizygous VOUS SNVs (Table 2).

There were three fetuses with positive NIPT results, and the suspected CNVs were verified by SNP array, including two likely pathogenic CNVs (dup 7p22.3q36.3, del 10q26.13q26.3) in two cases and a VOUS CNV (dup 8p23.1) in one case. In addition, trio-CES identified a hemizygous, likely pathogenic variant in the *AFF2* gene (NM_002025: c.1262+1G>A) in the case of 8p23.1 duplication (Case #2).

In the six fetuses with ultrasonic soft markers, CMA revealed a pathogenic CNV in one case with choroid plexus cyst (del 2q36.3q37.3), likely pathogenic CNVs in five fetuses with increased NT (del 7q32.3q36.3, Case #4), a choroid plexus cyst (del Xp22.33), lateral ventricle dilation (del 17p13.3, Case #7), and an abnormal nasal bone (dup 22q11.21, del Xp22.33). Meanwhile, trio-CES identified a de novo, likely pathogenic variant in the *PRRT2* gene (NM_145239: c.649dupC, p.R217Pfs*8) in case #7. The reported VOUS in the *TRPS1* gene (NM_014112: c.422C>T, p.P141L) in case #4 was predicted to be deleterious by commonly used pathogenicity evaluation algorithms.

Of the 11 fetuses with ultrasonographic diagnoses with structural abnormalities, pathogenic CNVs were detected in seven cases, including two cases with cardiac defects (del 11q24.1q25, del 1p36.33p36.31 in), 2 fetuses with a right aortic arch (del 16p13.3, del 22q11.21), and 3 fetuses with renal dysplasia (del 7q11.23, del 17q12); likely pathogenic CNVs were detected in 2 fetuses with cardiac defects or multiple anomalies (del 4q33q35.2, del 12p13.33p13.32). VOUS CNVs were detected in two fetuses with cardiac defects or fetal growth restriction (dup Xq26.1q26.3, dup 8p23.1). In addition, trio-CES identified likely pathogenic variants in two fetuses with compound heterozygous variants in the *CEP152* and *MYH2* genes (Case #10, #16) and a hemizygous VOUS in the *SLX4* gene due to its location at the region 16p13.3 deletions (Case #14).

### 3.3. CNV-Seq versus SNP Array Analysis

The CNV detection resolution for both CNV-seq and SNP array analysis were at the 100 kb level. The clinically significant CNVs originally identified by SNP-array analysis were all detected by CNV-seq. The size of the CNVs detected by CNV-seq ranged from 199 kb to 159,114 kb, whereas they ranged from 195 kb to 159,076 kb by SNP array analysis (Table 1). Overall, the sizes of CNVs detected by CNV-seq were slightly larger (100.9% on average) than that of the SNP array results.

**Figure 1:**
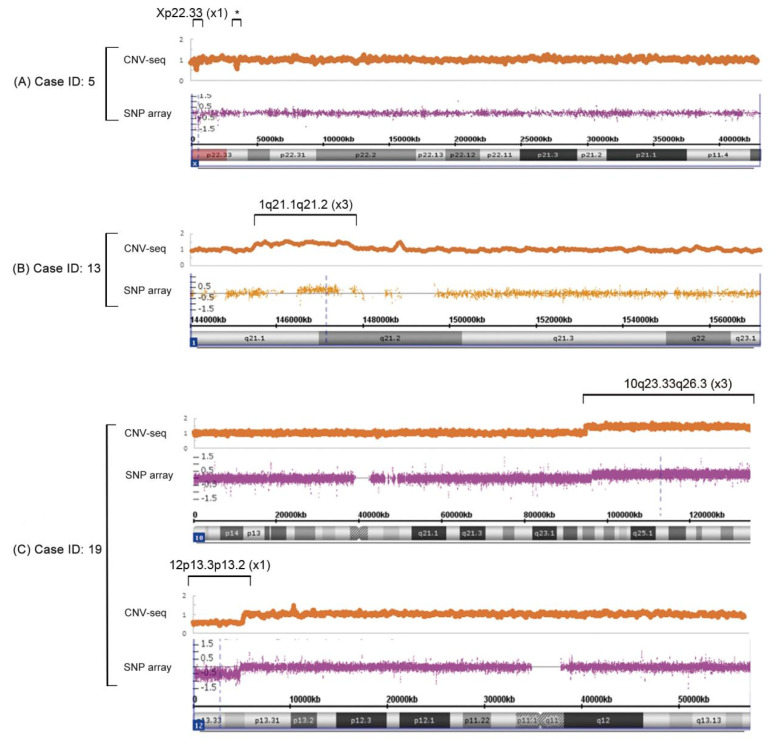
Comparison of CNVs detected by CNV-seq and SNP array. (**A**) In case #5, a deletion at Xp22.33 (×1) was detected by both CNV-seq and SNP array. * Another likely benign copy number variation Xp22.33 (×1) was also detected in this region by CNV-seq, and the length was about 125 kb. This variant was not detected by SNP array, probably due to the lack of SNP probes in this region. (**B**) Prenatal case #13 had a duplication at 1q21.1q21.2 (×3). The size detected by CNV-seq was significantly larger than that by SNP array. (**C**) Both CNV-seq and SNP array detected a deletion at 12p13.33p13.32 (×1) and a duplication at 10q23.33q26.3 (×3) in prenatal case #19.

The 195 kb deletion in Xp22.33 in case #5 was the smallest CNV detected by SNP-array analysis and was identified as a 199 kb deletion by CNV-seq (Figure 1A). The biggest size difference between the SNP array and CNV-seq was about 2.36 Mb in case #13, which was a 1.33 Mb gain by SNP array or a 3.69 Mb gain by CNV-seq (Figure 1B). In case #19, a 10q23q26 duplication and a 12p13 deletion were detected by CNV-seq, and the breakpoints were very close to the SNP array analysis (Figure 1C). Four mosaic CNVs in three cases were identified by both CNV-seq and SNP array analysis, and CNV-seq quantitated mosaicism at levels between 40–66% by the read-depth information. The level of moisacism inferred from the SNP-array was 10–30% in the same cases.

## 4. Discussion

In the prenatal setting, clinically significant CNVs are commonly classified as pathogenic, likely pathogenic, or VOUS [30,31]. Pathogenic CNVs cause a well-defined phenotype in a substantial proportion of known syndromes (e.g., 1p36 deletion, 22q11.21 deletion), which are recurrent in fetuses with congenital anomalies. In this case series, pathogenic CNVs were detected in six fetuses with ultrasonic structure anomalies and one fetus with a choroid plexus cyst (del 2q36.3q37.3, case #6). Chromosome 2q37 deletion syndrome (MIM# 600430) is characterized by mild-to-moderate developmental delay, short stature, hypotonia, autism spectrum disorder, and congenital heart disease [32,33,34]. The case with the 2q36.3q37.3 deletion did not present with the associated phenotypes mentioned above. In this case series, the detection rate of likely pathogenic CNVs was 45% (9/20), pathogenic CNVs was 40% (8/20), and VOUS CNVs was 15% (3/20). The detection rate of likely pathogenic SNVs was 20% (4/20), and VOUS SNVs was 10% (2/20). Among them, some cases have many variants, and the principle of statistics is to count only one of the variants according to the clinical manifestations.

Likely pathogenic CNVs often are characterized by variable phenotypes and/or incomplete penetrance and are associated with a rather unpredictable phenotype that does not present prenatally (e.g., intellectual disability, epilepsy). For example, the 22q11.2 duplication syndrome (MIM# 608363) has reduced penetrance and variable expressivity, from seemingly asymptomatic to severe phenotypes, including intellectual disability, growth retardation, muscular hypotonia, cardiac anomalies [35,36]. In this study, a de novo 22q11.2 duplication was detected in case #8 with an abnormal nasal bone, and no known pathogenic/likely pathogenic exonic SNVs/InDels were identified by trio-CES, which has brought some difficulties and challenges to providing genetic counselling.

CNVs that cannot be classified as benign, pathogenic, or likely pathogenic were designated VOUS, including a minority of novel CNVs and only a limited number of reported CNVs. Nowadays, whether VOUS should be reported in the prenatal setting is a controversial issue at different medical centers [19,22,37]. Many clinicians believe that uncertain results may cause anxiety in pregnant women and an ‘unnecessary’ termination of pregnancy in some cases. In prenatal diagnosis, it is inevitable to detect variants of uncertain clinical significance due to the much higher resolution of molecular diagnostic techniques such as SNP array and NGS platforms. However, a result of uncertain significance still offers information to clinicians. Clinical cytogeneticists can predict the pathogenicity of some uncertain variants by utilizing data reported in other individuals, whether they are inherited or de novo. In this study, VOUS CNV/InDels were reported in six cases, including three fetuses with two or more CNVs.

Types of germline genomic variants include chromosome aneuploidies, micro-deletion/duplication, intragenic CNVs, and SNV/InDels. Two or more variant types may occur in a subject. A number of studies have indicated that pathogenic or likely pathogenic CNVs and SNV/InDels can be identified concurrently in a pediatric patient [12,13,14,15]. It is known that the diagnosis of genetic diseases in patients is mainly based on the clinical phenotypes. However, as opposed to postnatal assessment of defined clinical features, the recognition of fetal phenotypes is largely dependent on the obstetric ultrasonographic examination. In addition, factors leading to variable prenatal clinical presentation include the proficiency of personnel, imaging approaches, and the stage of gestation by prenatal ultrasonography. Some clinical phenotypes involving organ dysfunction, such as intellectual disability, metabolic disturbance, and deafness, may not be recognized in the prenatal period. The role of prenatal diagnosis is to provide prospective parents with appropriate counseling for an informed choice about the ongoing pregnancy. The implication might also concern prospective postnatal management and future reproductive decisions. Therefore, it is essential to provide a comprehensive analysis of genetic variants and their correlations with the often limited or uncertain fetal phenotype. As such, it is essential to provide a comprehensive analysis of genetic variants for fetuses with ultrasonic abnormalities due to limited or uncertain fetal phenotypes.

As a sensitive microarray technology, SNP array permitted the detection of triploidy, chromosome aneuploidy, submicroscopic CNVs, UPD, and chromosome mosaicism with a level above 30%, which has been recommended as a first-tier test for the detection of CNVs in pediatric patients with developmental delays and fetuses with ultrasonic anomalies [3,38,39]. Recently, CNV-seq based on NGS is emerging as a high-resolution technology (100 kb) for genome-wide CNV detection, which has the advantages of relatively low cost and smaller sample size compared to CMA [23,40]. It has been reported that CNV-seq can detect chromosome mosaicism with a level above 20% [41]. In this case series, CNVs detected by the SNP array were all identified by CNV-seq, including mosaicism in four cases. A results comparison of the two different technologies indicated that the capacity of CNV detection is similar.

From the perspective of the accuracy of CNV detection, CMA is the first-line clinical technology with high accuracy, but it is also expensive. Low-pass WGS CNV detection is also relatively reliable. If medical exons can also be further used for CNV detection, from the perspective of health economics, the future trend of a combination of prenatal genetic testing technology may include CNV-seq plus exome sequencing, which can simultaneously detect CNVs and monogenic diseases in a single experiment. In conclusion, our study demonstrated that microdeletion/duplication syndromes and monogenic disorders may co-exist in a subject, and CNVs may contribute to the uncovering of recessive disease alleles. The role of prenatal diagnosis is to provide prospective parents with appropriate counseling for an informed choice about the ongoing pregnancy. As a result, the application of a comprehensive genomic evaluation (CNVs and SNV/InDels) concurrently may be of great value in prenatal diagnosis. Therefore, it can be inferred from this study that CNV-seq+ES may be the main technical strategy for prenatal diagnosis of known genetic diseases for cases of fetal structural abnormalities in the future, in terms of health economics and the effect of the simultaneous detection of CNVs and monogenic diseases. However, if research purposes such as the discovery of new genes need to be considered, then a combination of CNV-seq plus whole exome sequencng (WES) may be more appropriate.

## Figures and Tables

**Table 1:** Comparison of CNVs detected by CMA and CNV-seq in 20 prenatal samples.

Case ID	Fetal NIPT or Ultrasound Findings	CMA (SNP Array)			CNV-seq (~ 0.1 × GS)
		CNVs	Classification	Parental Origin	
1	NIPT positive: chromosome 7 duplication	arr7p22.3q36.3(43,376–59,119,707) × (2~3), Mosaic dup; 159.07 Mb	LP	Unknown	seq[GRCh37/hg19](10,003–159,125,000) × 2.48, Mosaic dup; 159.11 Mb
2	NIPT positive: chromosome 8 partial duplication for 6.66 Mb	arr8p23.1(6,999,219–11,898,980) × 3; 4.89 Mb	VOUS	Unknown	seq[GRCh37/hg19](6,160,003–12,185,000) × 3; 6.02 Mb
3	NIPT positive: chromosome 10 partial deletion	arr10q26.13q26.3(125,920,216–135,427,143) × 1; 9.50 Mb	LP	Unknown	seq[GRCh37/hg19](125,910,003–135,460,000) × 1; 9.54 Mb
4	Increased nuchal translucency (NT = 5.0 mm)	arr2p25.3p24.1(12,770–22,097,178) × (2~3), Mosaic dup; 22.08 Mb	VOUS	Unknown	seq[GRCh37/hg19](10,003–21,510,000) × 2.4, Mosaic dup; 21.49 Mb
		arr7q34q36.3(138,304,582–159,119,707) × 1; 20.81 Mb	LP	Unknown	seq[GRCh37/hg19](138,350,003–159,125,000) × 1, 20.77 Mb
		arr7q32.3q33(131,549,515–135,949,659) × (1~2), Mosaic del; 4.40 Mb	LP	Unknown	seq[GRCh37/hg19](132,400,003–138,350,000) × 1.5, Mosaic del; 5.94 Mb
5	Fetal bilateral choroid plexus cyst	arrXp22.33(484,176–679,369) × 1, Female; 0.19 Mb	LP	Unknown	seq[GRCh37/hg19](519,824–719,821) × 1; 0.19 Mb
6	Maternal serologically screening positive (1/71), left choroid plexus cyst	arr2q36.3q37.3(228,800,365–240,549,686) × (1~2), Mosaic del; 11.74 Mb	P	De novo	seq[GRCh37/hg19](228,577,003–240,532,000) × 1.34, Mosaic del; 11.95 Mb
7	Fetal bilateral lateral ventricle dilation (left 11 mm/right 13 mm)	arr17p13.3(525–1,570,118) × 1; 1.56 Mb	LP	Unknown	seq[GRCh37/hg19](3–1,550,000)x1; 1.54 Mb
8	Maternal serologically screening positive, absence of nasal bone	arr22q11.21(18,640,729–21,465,659) × 3;2.82 Mb	LP	De novo	seq[GRCh37/hg19](18,725,003–21,650,000) × 3; 2.92 Mb
9	The angle of the nasal bone on the frontal extension line is small, and the fetal nasal bone is flat	arrXp22.33(1,240,319–2,696,690) × 1, Female; 1.45 Mb	LP	Unknown	seq[GRCh37/hg19](1,586,501–2,710,000) × 1, Female; 1.12 Mb
		arrXp22.33(2,696,692–3,185,613) × 0, Female; 0.48 Mb	LP	Unknown	seq[GRCh37/hg19](2,710,003–3,160,000) × 0, Female; 0.44 Mb
10	Increased NT (4.4 mm), single ventricle, absence of nasal bone	arr4q33q35.2(171,020,317–190,957,473) × (1~2), Mosaic del; 19.93 Mb	LP	De novo	seq[GRCh37/hg19](171,782,603–190,882,600) × 1.4, Mosaic del; 19.09 Mb
11	Increased NT (4.2 mm), fetal ventricular septal defect, left heart dysplasia	arr2p25.3p23.2(12,770–29,476,625) × 3; 29.46 Mb	VOUS	Unknown	seq[GRCh37/hg19](10,003–29,410,000) × 3; 29.39 Mb
		arr11q24.1q25(123,409,884–134,938,470) × 1; 11.52 Mb	P	Unknown	seq[GRCh37/hg19](123,417,003–134,942,000) × 1; 11.52 Mb
12	Right fetal atrium enlarged, the tricuspid valve was deformed downward, and the tricuspid regurgitation (mild)	arr1p36.33p36.31 (849,466–6,927,026) × 1; 6.07 Mb	P	Unknown	seq[GRCh37/hg19](885,003–7,035,000) × 1; 6.14 Mb
13	Fetal ventricular septal defect (4.2 mm)	arr1q21.1q21.2(146,488,131–147,819,294) × 3; 1.33 Mb	VOUS	Paternally Inherited	seq[GRCh37/hg19](145,885,003–149,585,000) × 3; 3.69 Mb
14	Fetal aortic valve constriction, right aortic arch, fetal gallbladder enlargement	arr16p13.3(3,055,930–6,711,356) × 1; 3.65 Mb	P	De novo	seq[GRCh37/hg19](2,635,003–6,710,000) × 1; 4.07 Mb
15	Right aortic arch with mirrored branches, U-annulation	arr22q11.21(18,644,790–21,465,659) × 1, Female; 2.82 Mb	P	De novo	seq[GRCh37/hg19](18,900,003–20,200,000) × 1, Female; 1.29 Mb
		arrXp22.33(2,058,612–3,245,042) × 1, Female; 1.18 Mb	LP	Maternally Inherited	seq[GRCh37/hg19](2,135,003–3,235,000) × 1, Female; 1.09 Mb
16	Right renal polycystic dysplasia, bilateral renal echo enhancement	arr7q11.23(72,723,370–74,154,209) × 1; 1.43 Mb	P	De novo	seq[GRCh37/hg19](72,650,003–74,150,000) × 1; 1.49 Mb
17	Right polycystic renal dysplasia, bilateral renal echo enhancement	arr17q12(34,822,465–36,307,773) × 1; 1.48 Mb	P	De novo	seq[GRCh37/hg19](34,500,003–36,275,000) × 1; 1.77 Mb
18	Fetal bilateral renal dysplasia, bilateral renal echo enhancement, left hydronephrosis, small right kidney	arr17q12(34,822,465–36,418,529) × 1; 1.59 Mb	P	De novo	seq[GRCh37/hg19](34,575,003–36,325,000) × 1; 1.74 Mb
19	Fetal growth restriction, Ventricular deficiencies, left renal polycystic dysplasia, right renal cortical enhancement.	arr10q23.33q26.3(96,180,463–135,427,143) × 3; 39.24 Mb	VOUS	Paternally Inherited (balanced translocation)	seq[GRCh37/hg19](96,150,002–135,400,000) × 3; 39.24 Mb
		arr12p13.33p13.32(173,786–4,843,935) × 1; 4.67 Mb	LP		seq[GRCh37/hg19](150,002–4,850,000) × 1; 4.69 Mb
20	Fetal growth restriction	arrXq26.1q26.3(130,063,290–133,719,843) × 2, Male; 3.65 Mb	VOUS	Unknown	seq[GRCh37/hg19](130,060,003–133,735,000) × 2, Male; 3.67 Mb

CNVs: copy number variants; VOUS: variants of unknown significance; P/LP, pathogenic/likely pathogenic.

**Table 2:** The clinical significance of comprehensive genetic variants in 6 fetuses.

Case ID	Fetal Ultrasound Findings	CNV Detection				Trio-CES			
		CNVs	Classification	Parental Origin	Disease	Variants; Inherited Pattern	Parental Origin	Classification	Disease
2	NIPT positive: chromosome 8 partial duplication for 6.66 Mb	8p23.1, Dup (×3)	VOUS	Unknown	8p23.1 duplication related disease	*AFF2* [NM_002025]: c.1262+1G>A, Hemi, Male; XL	Maternally inherited	LP	Mental retardation
4	Increased nuchal translucency (NT = 5.0 mm)	2p25.3p24.1, Mosaic Dup (×2.48)	VOUS	Unknown	2p25.3p24.1 duplication related disease	*TRPS1* [NM_014112]: c.422C>T (p.P141L), Het; AD	De novo	VOUS	Trichorhinophalangeal syndrome
		7q34q36.3, Del (×1)	LP		7q34q36.3 deletion related disease				
		7q32.3q33, Mosaic Del (×1.5)	LP		7q32.3q33 deletion related disease				
7	Fetal bilateral lateral ventricle dilation (left 11 mm/right 13 mm)	17p13.3, Del (×1)	LP	Unknown	17p13.3 microdeletion syndrome	*PRRT2* [NM_145239]: c.649dupC (p.R217Pfs*8), Het; AD	De novo	LP	PRRT2 related Episodic kinesigenic dyskinesia
10	Increased NT (4.4 mm), single ventricle, absence of nasal bone	4q33q35.2, Mosaic Del (×1.4)	LP	De novo	4q33q35.2 deletion related disease	*CEP152* [NM_001194998]: c.3629T>A (p.I1210N), c.1060C>T (p.R354 *), Compound Het; AR	Biparental inherited	VOUS; LP	Primary Microcephaly /Seckel syndrome
14	Fetal aortic valve constriction, right aortic arch, fetal gallbladder enlargement	16p13.3, Del (×1)	P	De novo	16p13.3 deletion syndrome	*SLX4* [NM_032444]: c.553G>A (p.D185N), Hemi ^#^; AR	Maternally inherited	VOUS	Fanconi anemia
16	Right renal polycystic dysplasia, bilateral renal echo enhancement	7q11.23, Del (×1)	P	De novo	Williams-Beuren syndrome	*MYH2* [NM_001100112]: c.3403C>T (p.R1135W), c.5826+2C>T, Compound Het; AR	Biparental inherited	VOUS; LP	Proximal myopathy and ophthalmoplegia

*: It was a frameshift mutation caused by the insertion of another cytosine after nine cytosines, which resulted in the change of amino acid sequence at position 217 Arg and other amino acid sequences, and premature termination at position 224; #: The SLX4 gene is located at the region 16p13.3 deletion. CES: clinical exome sequencing; CNVs: copy number variants; VOUS: variants of unknown significance; P/LP, pathogenic/likely pathogenic; AD: dominant inheritance; AR: recessive inheritance; XL, X-linked.

## Data Availability

The data that support the findings of this study are available from the corresponding author upon reasonable request.
